# Descriptive epidemiology of measles cases in Bauchi State, 2013–2018

**DOI:** 10.1186/s12889-021-11063-6

**Published:** 2021-07-05

**Authors:** Polycarp Uchechukwu Ori, Ayo Adebowale, Chukwuma David Umeokonkwo, Ugochukwu Osigwe, Muhammad Shakir Balogun

**Affiliations:** 1 Nigerian Field Epidemiology and Laboratory and Training Programme, Abuja, Nigeria; 2 Department of Epidemiology and Medical Statistics, Faculty of Public Health, University of Ibadan, Ibadan, Nigeria; 3 Department of Community Medicine, Alex Ekwueme Federal University Teaching Hospital, Abakaliki, Ebonyi State Nigeria; 4 Africa Field Epidemiology Network, Abuja, Nigeria

**Keywords:** Measles, Vaccination, Mortality, Bauchi state

## Abstract

**Background:**

Measles accounts for high morbidity and mortality in children, especially in developing countries. In 2017, about 11,190 measles cases were recorded in Nigeria, including Bauchi State. The aim of this study was to describe the trend and burden of measles in Bauchi State, Nigeria.

**Method:**

We analyzed secondary data of measles cases extracted from the Measles Surveillance data system in Bauchi State from January 2013 to June 2018. The variables extracted included age, sex, doses of vaccination, case location and outcome. Data were analyzed using descriptive statistics, logistic regression, and multiplicative time series model (α = 0.05).

**Results:**

A total of 4935 suspected measles cases with an average annual incidence rate of 15.3 per 100,000 population and 57 deaths (Case Fatality Rate, CFR: 1.15%) were reported. Among the reported cases, 294 (6%;) were laboratory-confirmed, while clinically compatible and epi-linked cases were 402 (8%) and 3879 (70%), respectively. Of the 4935 measles cases, 2576 (52%) were males, 440 (9%) were under 1 year of age, and 3289 (67%) were between 1 and 4 years. The average annual incidence rate among the 1–4 year age-group was 70.3 per 100,000 population. The incidence rate was lowest in 2018 with 2.1 per 100,000 and highest in 2015 with 26.2 per 100,000 population. The measles cases variation index per quarter was highest in quarter 1 (198.86), followed by quarter 2 (62.21) and least in quarter 4 (10.37) of every year. Only 889 (18%) of the measles cases received at least one dose of measles vaccine, 2701 (54.7%) had no history of measles vaccination while 1346 (27.3%) had unknown vaccination status. The fatality of measles in Bauchi State were significantly associated with being under 5 years (AOR = 5.58; 95%CI: 2.19–14.22) and not having at least a dose of MCV (OR = 7.14; 95%CI: 3.70–14.29).

**Conclusion:**

Measles burden remains high in Bauchi State despite a decrease in its incidence over the study years. Most of the cases occurred in the first quarter of every year. Improved routine measles surveillance for prompt case management could reduce the burden of the disease in Bauchi State.

## Background

Measles remains a significant cause of morbidity and mortality among young children, globally. Despite that measles is a vaccine preventable disease, its outbreak is recorded mostly between February and April of every year, which coincides with the dry season [1]. In 2016, about 39.9 million cases of measles were recorded with 777,000 deaths, worldwide. Africa and Southeast Asia account for 70 and 84% of cases of measles and measles-related mortality reported, respectively, worldwide [2–4]. The burden of measles remains high in Nigeria, the most populous country in Africa. Measles are endemic in most states in the northern Nigeria, including Bauchi State [2, 5–7]. In 2012, five WHO regions, including Africa, agreed and adopted implementation of five core strategies aimed at eliminating measles by 2020 [8–10]. Progress in achieving the goal was evaluated in 2015, and there was a need to focus more on strengthening immunization activities [8]. Although vaccination has significantly led to reduction of 73% of global deaths from measles between 2000 and 2018, the burden of measles remains high in Nigeria [2, 5–7] More than three - quarters of measles-related deaths are recorded in countries with poor development, low earnings per capita, and the weak health system recorded [2, 11]. Malnutrition, poor case management, lack of immunization and overcrowding are common factors associated with measles mortality [2, 11–13]. These factors are common in a low-income country such as Nigeria.

In 2012, the African Region endorsed Global Measles Mortality Prevention Strategies, and began the implementation of the recommended strategies by setting a target to be achieved by the year 2020 [11, 13]. The strategies aimed at improving the measles-containing vaccine (MCV1) coverage and measles case management. Developing case-based surveillance of all suspected measles cases with laboratory confirmation remained one of the objectives in achieving the aim [11, 12, 14]. On successful implementation of the strategies, the expected outcome was to achieve a 93% reduction in the incidence of measles, and a 92% reduction in the African region’s reported death rates [13, 14].

Active measles case-based surveillance system is one of the epidemiological methods designed to achieve the reduction in measles cases and mortality associated with the disease. The surveillance system aids in identifying the measles etiology, determining the extent of the disease, studying its progression, and evaluating its preventive and therapeutic measures. The outcome of the surveillance system guides the development of public health policy on measles. Although Nigeria has intensified case-based surveillance of measles cases, little is known on the trend and burden of the cases, especially in the northeast part of the country. This study was therefore conducted to describe the burden of measles and to determine factors associated with fatality among suspected measles cases in Bauchi State from 2013 to 2018.

## Method

### Study design

We analyzed case-based surveillance data of measles as reported in Bauchi State from 2013 to 2018. Bauchi State occupies a total land area of 49,119 km^2^, representing about 5.3% of Nigerian landmass. The estimated population of Bauchi State in 2015 was 6,275,523 based on 2006 census when the population was 4,653,066 with growth rate of 3.6%. In 2015, the estimated population of children under 5 years was 1,255,105 based on the 2006 projection. The State comprises 20 local government areas (LGAs), and the State is predominantly inhabited by people of Fulani, Hausa, and the Geraway ethnic background. Islam is the main religion and, farming, livestock rearing, and trading are the primary occupations of the inhabitants [15]. The State has 1,046 health facilities that offer immunization services through, fixed routine immunization sessions and outreaches to rural and hard to reach communities. Three of these health facilities are tertiary health institutions, and 13 of the health facilities are private health facilities.

### Data collection

Data were collected from the Integrated Disease Surveillance and Response System (IDSR 002, weekly data) in Excel format from the Bauchi State Ministry of Health’s Epidemiology Unit. The Disease Surveillance and Notification Officers (DSNOs) from all the 20 local government areas routinely submit these data to the Bauchi State Ministry of Health. Health facilities report to LGA DSNOs daily using the standard case-based investigation form. After collection of data by DSNO, the variables in the data were recorded based on the data analysis requirements. The available variables in the surveillance system included age, sex, doses of vaccination, case classification, outcome, district of cases**,** location, and LGAs.

### Measles and outcome definitions

The suspected measles case was defined as any person with generalized maculopapular rash and fever together with any of the following: cough or coryza (runny nose) or conjunctivitis (red eyes). It could also be defined as any person suspected of measles by a clinician. A confirmed case could be confirmed by a laboratory, epidemiologically linked or clinically compatible. A laboratory-confirmed measles case included a suspected case of measles which is confirmed by detection of the serum antibody by enzyme-linked immunosorbent assay in the absence of measles vaccination within 1 month of specimen collection. An epidemiological case is a suspected case with no serological confirmation but is linked (in place, person and time) to a laboratory-confirmed measles case [16, 17]. The clinically compatible case is that which satisfies the clinical case features with no laboratory testing or epidemiological linkage [1].

A suspected case of measles is said to be a non-measles after it has been thoroughly investigated, including the collection of adequate blood specimens, and lacks serological evidence of recent infection with the measles virus (IgM Negative). Additionally, those suspected cases that are IgM positive as a result of measles vaccination within a month of blood specimen collection are considered as non-measles cases [18].

Measles death is any death from an illness that occurs within1 month of the onset of rash in a confirmed case of measles [6].

### Data analysis

Secondary data from 2013 to 2018 cases were analyzed using Epi Info 7 version and Microsoft Excel 2010. Denominators for person rate estimations were based on the 2006 Nigerian population census projections. Descriptive statistics were used to generate trend over the study period under review. To ascertain which quarter of the year experienced the highest number of reported cases of measles patients, we used a multiplicative time series model to examine the trend. The model is

1 $$ {y}_t={T}_t\times {S}_t\times {C}_t\times {I}_t $$

Where; *I*_t_ is the observed value of the time series in time t, T_t_ is the trend component in time t; S_t_ is the seasonal component in time t; C_t_ is the cyclical component in time t, and I_t_ is the irregular component in time t. If the parameters *C*_*t*_ and *I*_*t*_ are assumed to be constant and 1. Thus, by making *S*_*t*_ the subject, equation * becomes

2 $$ {S}_t={y}_t/{T}_t $$

Average seasonal variation by quarter (ASVQ) was estimated as:

$$ ASVQ=\frac{\sum_{i=1}^{n_i}\left(\left({y}_{t_i}/{T}_{t_i}\right)\times 100\right)}{n_i} $$

Where y_t is observed

$$ Variation\ index= ASVQ-\left(\frac{300-{\sum}_{j=1}^r\left[ ASVQ\right]}{r}\right), $$

Where r is the number of quarters.

We used bivariate analysis and logistic regression to determine factors associated with the outcome status of the reported measles cases (α =0.05).

### Ethical consideration

We obtained ethical approval from Bauchi State Ministry of Health with protocol number BSMOH/REC/23/2020 and approval number NREC/03/11/19B/2020/26. To ensure confidentiality of the subjects, we excluded all identifying information such as name and address from the study.

## Results

**Fig. 1:**
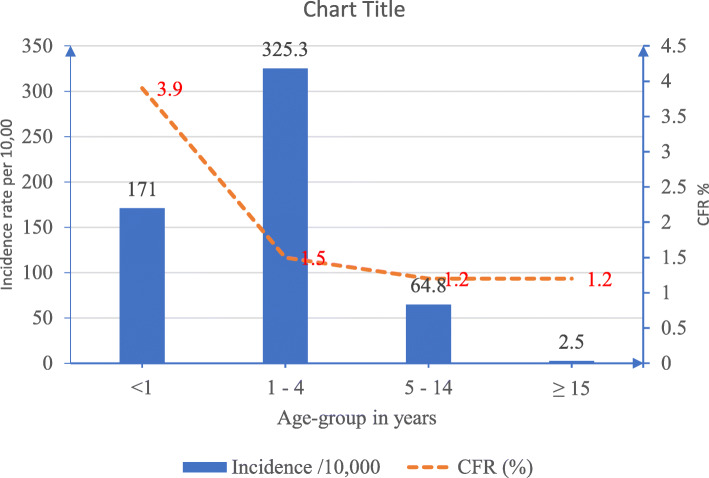
Age-specific incidence rate and CFR of measles cases in Bauchi, 2013–2018

A total of 4935 measles cases were reported in Bauchi State between January 2013 and June 2018. Cases showed pronounced variations in some socio-demographics variables (Table 1). Only 294 (6.0%) were laboratory-confirmed whereas most cases 3979 (80.6%) were clinically compatible measles. The median age for cases was 5 years while the interquartile range was 2–18 years. Fifty-two per cent of the suspected cases were males, 1–4 years old had most 3289 (66.0%) cases while least cases 76 (2.0%) aged at least 15 years. Most of the cases 3678 (75.0%) lived in rural areas. Interestingly, Bauchi LGA which is the state capital recorded the highest cases with 1576 (32%) of the cases (Fig. 1). More than half of the cases 2701 (55.0%) received at least one dose of measles vaccination while vaccination status for 1345 (27.0%) individuals were unknown (Table 1).

**Fig. 2:**
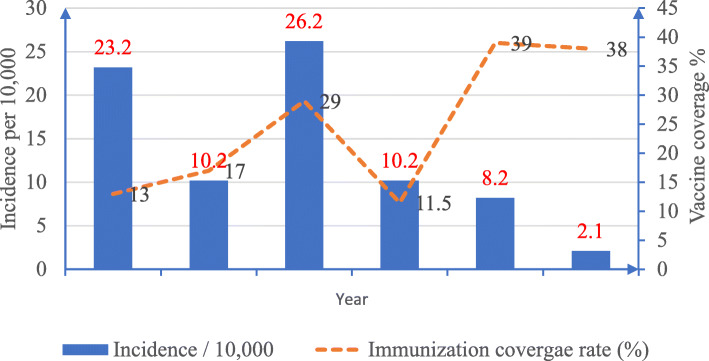
Incidence of measles and vaccination coverage in Bauchi State, 2013–2018

The total measles cases (laboratory-confirmed, clinically compatible case and epidemiologically linked measles cases) were 4575 (92.7%). Among the total measles cases, 57 deaths were recorded (CFR of 1.15%) (Fig. 2).

**Table 1:** Socio-Demographic Characteristics of Measles Cases in Bauchi, 2013–2018

Variables	Frequency (***N*** = 4936)	Percentage (%)
**Sex**		
Male	2567	52.0
Female	2369	48.0
**Age group (Years)**		
< 1	440	9.0
1–4	3289	66.0
5–14	1130	23.0
≥ 15	76	2.0
**Location**		
Urban	1258	25.0
Rural	3678	75.0
**Vaccination Status**		
0 Dose	889	18.0
≥ 1 Dose	1207	55.0
Unknown	1345	27.0

Figure 2 highlighted the relationship between burden of measles and age. Incidence of cases was highest among age-group 1–4 years with an incidence rate of 325.3 per 10,000 population. Age-group < 1 year had incidence of 171.3 per 10,000 population, and 2.5 per 10,000 population among ≥15 years. The CFR was 3.9% among < 1 year population and 1.5% among 5–14 years and ≥ 15 age-groups had CFR of 1.2%.

**Fig. 3:**
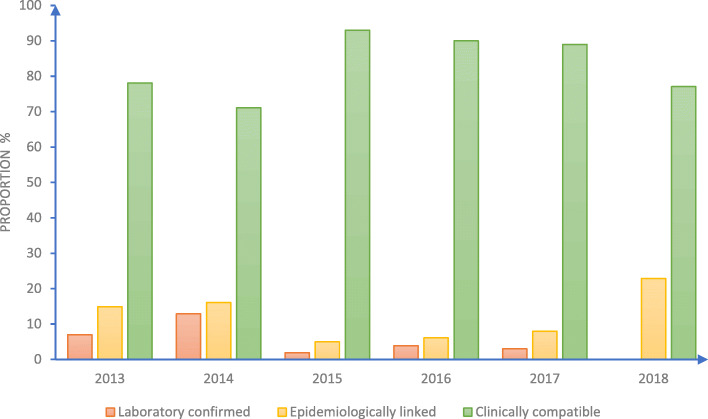
Yearly classification of measles cases in Bauchi, 2013–2018

In Fig. 3, we reported that the incidence of measles was highest in 2015 (26.2%) and least in 2018 (2.1%). Incidence of measles declined between 2013 and 2018 except for an upsurge observed in 2015. The available administrative vaccination coverage (Fig. 3) shows that the coverage was highest in 2015 (26.2%) and was followed closely by the year 2013 (23.2%) and least in 2018 (2.1%).

**Fig. 4:**
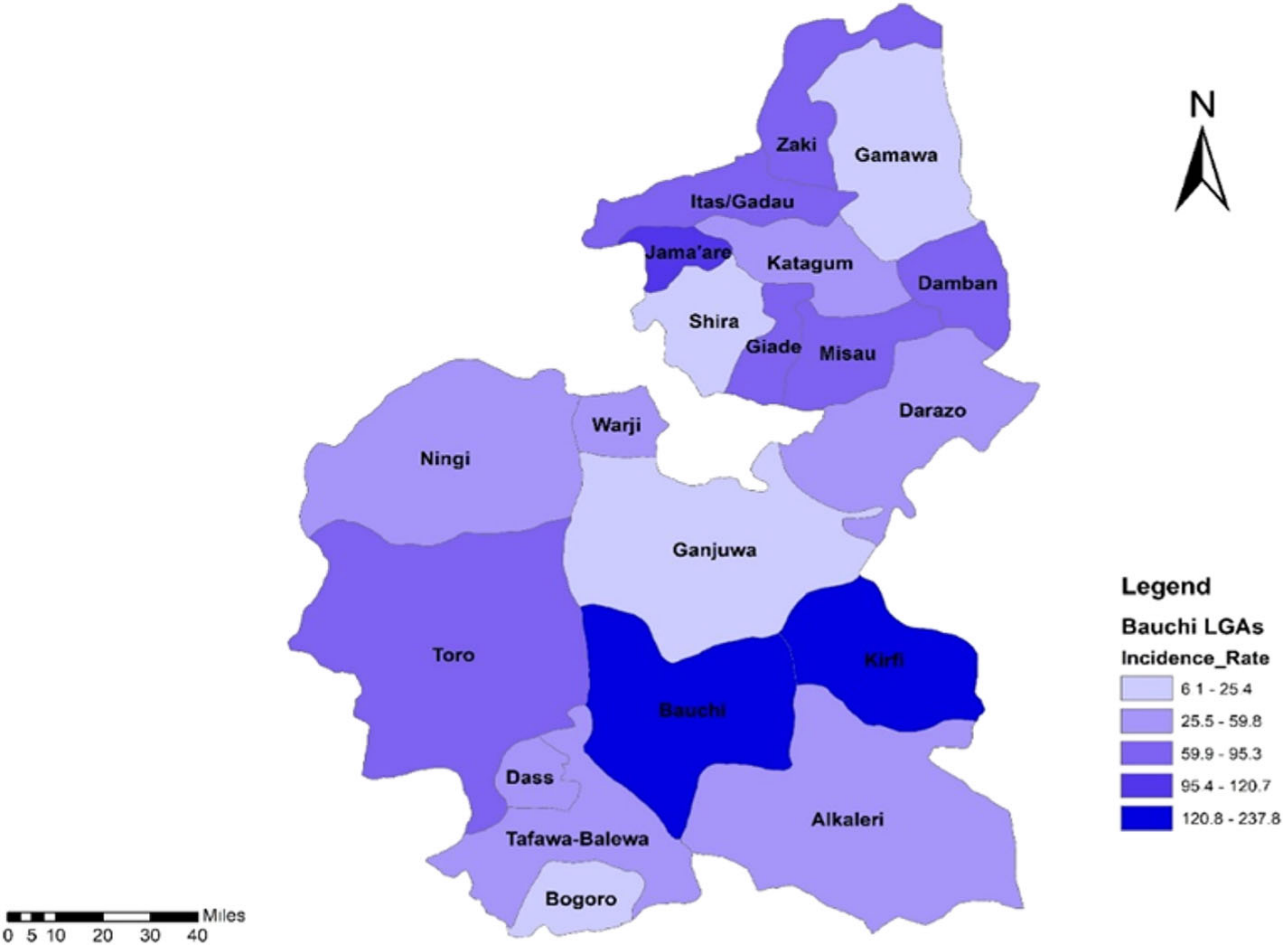
Incidence rate of measles case by LGAs in Bauchi State, 2013–2018

In Fig. 4, the data highlighted the classification and variation of confirmed measles cases across the 6 years. More than 70% of the confirmed cases were based on clinical compatibility with lowest (71%) in 2014 and highest in 2015. Less than 15% of confirmed cases were based on laboratory evidence with highest laboratory confirmation in 2017. In 2018, there no laboratory confirmation of any of the reported cases. The proportion of epidemiologically linked cases ranged from 5% in 2015 to 23% in 2018.

**Fig. 5:**
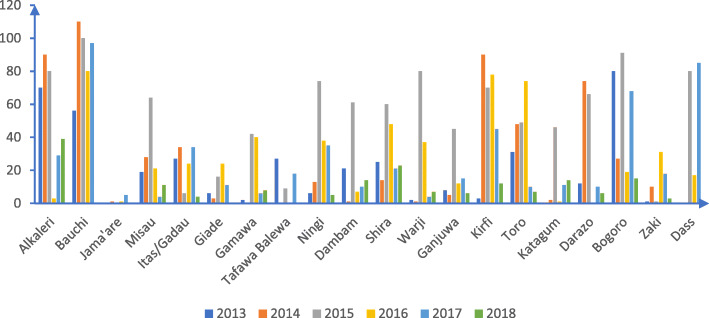
Trend of measles vaccine coverage per LGA in Bauchi State, 2013–2018 (administrative data)

Figure 5 shows the incidence of measles across the 20 LGAs in Bauchi State. Bauchi and Kirfi LGAs recorded measles of 120.8–237.8 per 100,000 population. Alkaleri, Ningi, Tafawa-Balewa, Dass, Warji had an incidence rate of 25.5–59.8 per 100,000 population while Bogoro, Shira, Ganjuwa and Gamawa LGAs measles incidence ranged from 6.1–25.4 per 100,000 population.

In Table 2, the identified determinants of mortality among suspected measles cases were age and vaccination status. Measles related death was significantly higher among individuals aged below 5 years compared to those who were 5 years and above, AOR 5.58 (95% C.I = 2.19–14.22). Those who live in rural areas are 2.2 times more likely to die of measles related death than those in urban areas, however, the difference in deaths between the two areas are not significant, (AOR 2.23, 95% CI = 0.94–5.29). The results further showed a significant higher mortality among those who have never received any dose of vaccination in contrast with those who received at least a dose of measles vaccine (AOR = 7.14, 95% C.I = 3.70–14.29).

**Table 2:** Factors associated with survival status among suspected measles cases in Bauchi, 2013–2018

Variables	Outcome		cOR (95% CI)	AOR (95% CI)
	Dead	Alive		
**Age (Years)**				
< 5	52	3677	3.39 (1.35–8.52)*	5.58 (2.19–14.22)*
≥ 5	5	1201		
**Sex**				
Male	25	2541	1.04 (0.6–1.76)	0.71 (0.41–1.23)
Female	32	2337		
**Location**				
Rural	51	3626	2.93 (1.25–6.85)*	2.23 (0.94–5.29)
Urban	6	1252		
**Vaccination Status**				
0 dose	45	844	5.33 (2.8–10.14)*	7.14 (3.70–14.29)*
≥ 1 dose	12	1200		

*Significant at *p* < 0.05

**Fig. 6:**
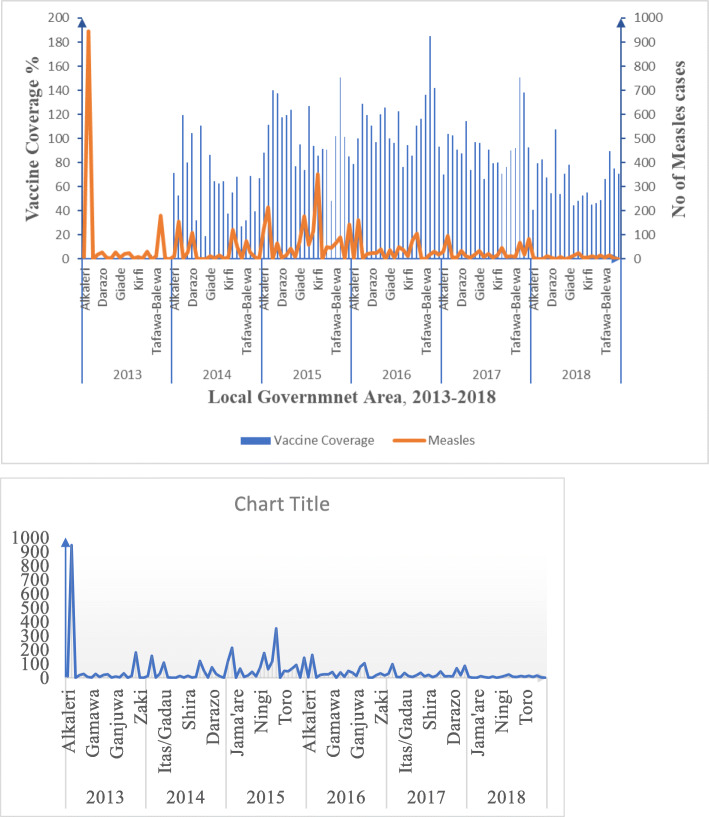
Trend of measles vaccination proportion with measles per year per LGA in Bauchi State, 2013–2018

The data, as presented in Fig. 6, shows the cumulative measles vaccination coverage for each local government area for the period under review.

**Fig. 7:**
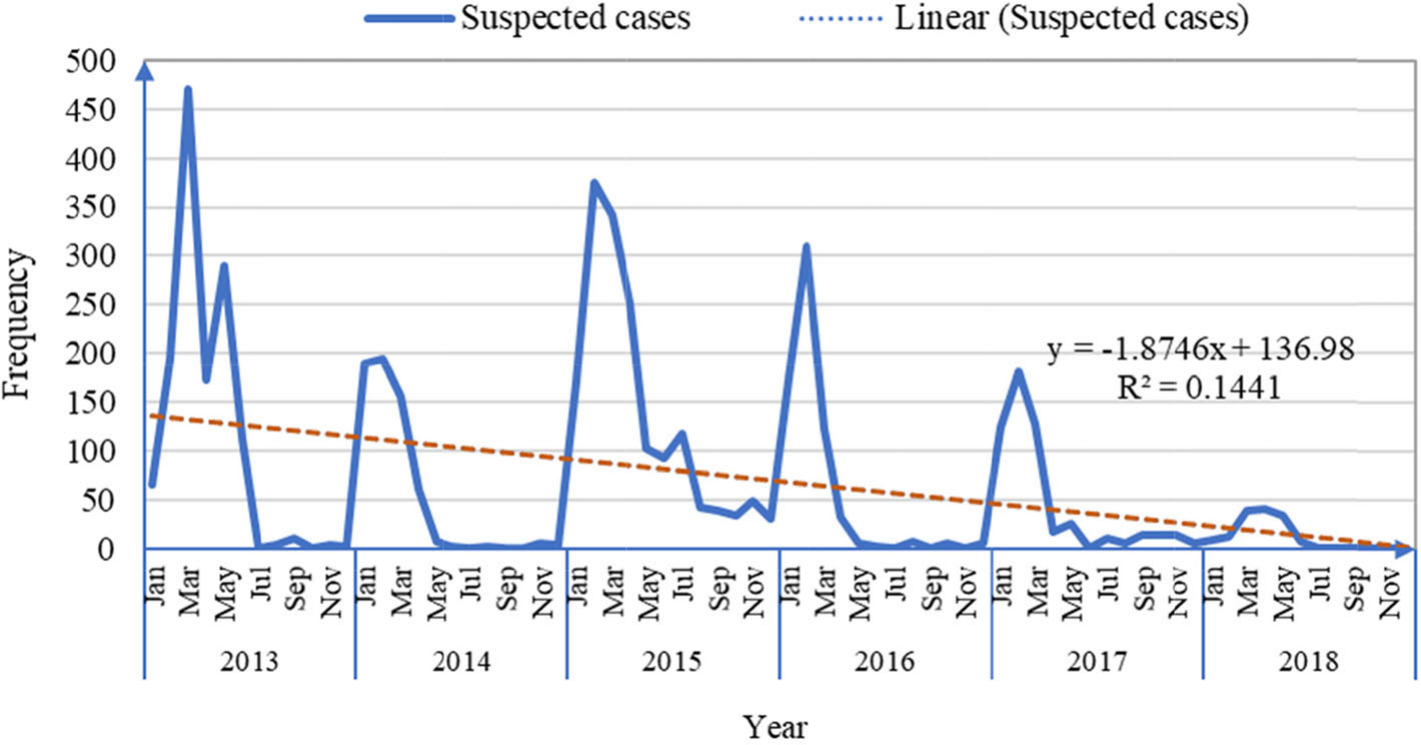
Trend in the distribution of suspected measles cases by months in Bauchi State, 2013–2018

As highlighted in Fig. 7, The data indicate that the suspected number of measles cases was highest between January and April of every year and least experienced from July to November. The trend line shows a downward trend in the suspected cases between 2013 and 2018 with the slope being − 1.8746. The trend indicates a reduction in the number of suspected cases of measles in the study period.

Using multiplicative model, the quarterly variation in the number of suspected cases of measles were presented in Table 3. The monthly suspected measles cases were aggregated using 3 months moving average method, and the outcome is shown in column 5 of the Table 3. The seasonal variation in the data was also shown in column 5 of Table 3. Although cases were recorded throughout the year, most cases occurred in the first quarter of a year (January – March), the peak of the dry season in the study area. After the first quarter, cases decreased down the third and last quarter of the year.

**Table 3:** Multiplicative model of seasonal variation in the number of suspected cases of measles in Bauchi State, 2013–2018

Year	Quarter	Quarterly number of cases (***y***_***t***_)	3 Months Moving Total	3 Months Moving Average (***T***_***t***_)	Seasonal Variation (***y***_***t***_/***T***_***t***_) ***×*** 100
2013	1	730			
	2	575	1320	440.00	130.68
	3	15	596	198.67	7.55
	4	6	561	187.00	3.21
2014	1	540	616	205.33	262.99
	2	70	613	204.33	34.26
	3	3	82	27.33	10.98
	4	9	900	300.00	3.00
2015	1	888	1344	448.00	198.21
	2	447	1532	510.67	87.53
	3	197	756	252.00	78.17
	4	112	917	305.67	36.64
2016	1	608	760	253.33	240.00
	2	40	656	218.67	18.29
	3	8	60	20.00	40.00
	4	12	453	151.00	7.95
2017	1	433	487	162.33	266.74
	2	42	504	168.00	25.00
	3	29	103	34.33	84.47
	4	32	121	40.33	79.34
2018	1	60	172	57.33	104.65
	2	80	140	46.67	171.43
	3	0	80	26.67	0.00
	4	0	0	0.00	

Table 4 shows variation index per quarter based on the decomposition of the variation observed by quarter shows that the largest proportion of measles cases reported in Quarter 1 (198.86), followed by quarter 2 (62.21) and least in quarter 4 (10.37) of every year.

**Table 4:** Decomposition of seasonal variation by the quarter

Year	Quarter			
	1	2	3	4
2013	–	130.68	7.55	3.21
2014	262.99	34.26	10.98	3.00
2015	198.21	87.53	78.17	36.64
2016	240.00	18.29	40.00	7.95
2017	266.74	25.00	84.47	79.34
2018	104.65	171.43	0.00	–
**Total**	1072.59	467.19	221.17	130.14
**ASVQ**	214.52	77.87	44.23	26.03
**Shared**	−15.66	−15.66	−15.66	−15.66
**Variation index**	**198.86**	**62.21**	**28.57**	**10.37**

## Discussion

Measles remains a significant cause of childhood mortality and morbidity in Nigeria, especially in the northern part of the country. We analyzed the surveillance data to describe the trend and burden of measles in Bauchi State, Nigeria. The reported measles cases in Bauchi state were high within the period under study. The high reported cases over study years were unexpected considering the access to free and safe measles vaccination in Nigeria during routine and supplementary immunization campaigns. Our result is incongruent with measles control because the ultimate global objective is to eradicate measles [2, 11]. Measles control is the initial part in measles eradication program which involves a reduction in morbidity and mortality. Several African countries, including Nigeria, are in the phase of measles control [19, 20].

In our findings, about four-fifths of the cases of measles occurred among the under-five children (Under-5). This finding is consistent with studies previously conducted in Nigeria and some other countries in sub-Saharan Africa [13, 21, 22]. Although measles affects all age groups in any population, the severity of this disease is common among the Under-5 and spread through adolescent years. In addition, our analysis indicated that unvaccinated young children were at highest risk of measles and its complications, including death. The vaccination coverage against measles disease is still low in Bauchi state. The low coverage may have accounted for the high incidence of measles recorded in the State over the periods under investigation. Lifetime immunity conferred by measles among the age groups of 5 years and above could be the possible explanation for lower incidence among the older age-group. Majority of the population aged 5 years and above might have received antigens through routine immunization or by previous measles infection. Other related prior studies in Nigeria, especially in the North-East region of the country where Bauchi State is located, have shown a high incidence of measles with the highest incidence in children aged between one and 5 years [1, 13, 23, 24]. The age-specific case fatality rate showed that Under-5 had significantly higher mortality than other age groups. Our finding did not show any significant difference between male and female in the incidence of measles. The finding is consistent with similar studies carried out in other Nigerian states [1, 13, 21, 24–29].

Interestingly, our study indicated that Bauchi LGA, the state capital, recorded highest although cases were recorded in all the LGAs in the State. The high incidence in the Bauchi LGAs could be due to overpopulation resulting from the displacement of people from neighbouring north-eastern states as a result of the insurgency and rural-urban migration. In addition, Urban slums which is common in the state capital where most of the young children are found could be another contributory factor to the high incidence of measles in the LGA. Also, the high sensitivity of the surveillance system in the Bauchi local government, the state capital, might be a possible explanation for a considerable number of cases found in the area. Similar findings to ours have been reported in some states in Nigeria [22]. Despite the relative high incidence of the cases in the urban LGA, deaths were higher in rural areas of the State than the urban settlements, though difference in death rates was not significant. Our finding is contrary to other related studies conducted in Nigeria where significant increase in CFR in rural areas than urban areas were reported [13, 21].

The CFR over the 6 years observed in this study was less than the 3–5% CFR recorded globally for developing countries (11.12) and higher than the 0.6% CFR for Nigeria [25]. For the 6 years, the rate of CFR decreased over the years, in line with the new rate in the reduction of measles mortality in Africa and worldwide [23–25, 30–33]. This finding could be attributed to improved surveillance, timely reporting of cases and effective clinical care as well as increased awareness of the importance of the routine immunization among caregivers, and knowledge of case management by health care workers. Given the time trend of the reported measles cases, most of the cases occurred in the first quarter of a year (January – March), the peak of the study area’s dry season. After March, the number of the reported cases then decreased down the third and last quarter of the year. This result is comparable to the trend recorded for measles in Nigeria and the WHO Africa region [30, 31, 34–36]. The increased incidence of measles coincides with the resumption time of children’s schools, which is the start of the dry season and a slow decline by the end of the year when the school is closed. These findings gave an insight into the time factor in planning various preventive measures, including routine and supplementary immunization.

Over the period under review, administrative vaccine coverage in all LGAs revealed that from 2015 to 2018, most LGAs achieved the 2019 Global Vaccine Action Plan target of at least 85% coverage. Expectedly, the incidence of measles had inverse relationship with vaccine coverage in all the LGAs across the time under review. However, administrative figures are frequently unreliable due to incomplete or incorrect primary documentation of vaccinations, errors in the compilation of monthly vaccine summaries, delayed or duplicated reporting and inaccurate population denominator estimates [37]. Based on the Nigeria National Immunization Coverage Survey (NICS) 2016/2017, the vaccination coverages of MCV in Bauchi (22.2%) over the study years were less than the corresponding national coverage (42%) [38]. There were a significantly higher incidence and CFR of measles cases among the unvaccinated population. The possible explanation for the rise in measles incidence in the unvaccinated population could be attributed to infection that occurs prior to the development of immunity, a maternal antibody intervention, or reduced efficacy of the vaccines due to inadequate handling and storage. Additionally, spatial and temporal variability in the immunity of populations and the weakening of immunity resulting from the measles vaccine over time could lead to a weakening of population immunity [1, 3, 24].

Our work is not without limitations; the reported data from the measles surveillance system in Bauchi state had some missing variables, which affected a more detailed description of the State’s burden of measles. Future studies on measles in Bauchi State could help to give a more comprehensive picture of measles in the state.

## Conclusion

Over the 6 years, the trend of measles in Bauchi State appears to decrease, but the burden remains relatively high. Under-5 years children are mostly affected by measles in the states, and case fatality was higher in this age group than every other segment of the population. While measles case fatality has been on the decline over the years under review, case confirmation from the laboratory was indeed low. Current case-based framework on measles eradication in Bauchi state should be strengthened, and more attention should be directed to immunization of Under-5. Enhanced measles surveillance and routine immunization system in Bauchi state are recommended.

## Data Availability

The data associated with report are available from the corresponding author upon reasonable request.

## Abbreviations

LGA: Local government area.
CFR: Case fatality rate
WHO: World health organization
SIA: Supplementary immunization activities
SV: Seasonal variation
QV: Quarterly variation

## References

[1] Aworabhi N, Numbere T, Balogun MS, Usman A, Utulu R, Ebere N, et al. Trends in measles cases in Bayelsa state, Nigeria: a five-year review of case-based surveillance data (2014–2018). BMC Public Health. 2020;20:938. 10.1186/s12889-020-09070-0. 10.1186/s12889-020-09070-0 PMC7296655 32539691

[2] Dabbagh A, Patel MK, Dumolard L, Gacic-Dobo M, Mulders MN, Okwo-Bele J-M (2016). Morbidity and Mortality Weekly Report Progress Toward Regional Measles Elimination-Worldwide, 2000–2016.

[3] Masresha BG, Braka F, Onwu NU, Oteri J, Erbeto T, Oladele S, et al. Progress Towards Measles Elimination in Nigeria: 2012 – 2016. J Immunol Sci. 2018;Suppl:135–9. PMID: 30957102; PMCID: PMC6446991. PMC6446991 30957102

[4] Progress towards measles elimination in Bangladesh, 2000–2016. Relev Epidemiol Hebd. 2017;92(29–30):405–12. 28737029

[5] Organización Mundial de la Salud. Global vaccine action plan 2011-2020. Geneva: World Health Organization; 2013.

[6] Tao W, Petzold M, Forsberg BC. Routine vaccination coverage in low- and middle-income countries: further arguments for accelerating support to child vaccination services. Glob Health Action. 2013;6:20343. 10.3402/gha.v6i0.20343. 10.3402/gha.v6i0.20343 PMC3643076 23639178

[7] Kagucia EW (2018). Health interventions to improve measles vaccination coverage and timeliness: an assessment of the immediate and long-term impact on vaccine-seeking in rural Kenya.

[8] Orenstein WA, Cairns L, Hinman A, Nkowane B, Olivé JM, Reingold AL (2018). Measles and rubella global strategic plan 2012–2020 midterm review report: background and summary. Vaccine..

[9] Thompson KM, Strebel PM, Dabbagh A, Cherian T, Cochi SL. Enabling implementation of the Global Vaccine Action Plan: developing investment cases to achieve targets for measles and rubella prevention. Vaccine. 2013;31 Suppl 2:B149–56. 10.1016/j.vaccine.2012.11.091. PMID: 23598476. 10.1016/j.vaccine.2012.11.091 23598476

[10] Mihigo R, Okeibunor J, Masresha B, Mkanda P, Poy A, Zawaira F, et. al. Immunization and vaccine development: Progress towards High and Equitable Immunization Coverage in the Africa Region. PMC6368246 30740602

[11] World Health Organization. Eliminating measles and rubella and preventing congenital rubella infection. Copenhagen: World Health Organization; 2005. p. 28.

[12] UNICEF. The State of the World’s Children 2017: Children in a Digital World. P1-40 UNICEF Division of Communication New York USA.

[13] Faruk AS, Adebowale AS, Balogun MS, Taiwo L, Adeoye O, Mamuda S (2020). Temporal trend of measles cases and impact of vaccination on mortality in Jigawa state, Nigeria, 2013-2017: a secondary data analysis. Pan Afr Med J.

[14] Ozawa S, Paina L, Qiu M (2016). Exploring pathways for building trust in vaccination and strengthening health system resilience. BMC Health Serv Res.

[15] Bauchi State Government. Bauchi State - Nigeria: Pearl of Tourism and Home of Hospitality. Available from: https://www.bauchistate.gov.ng/about-bauchi-state/. Accessed 14 May 2020.

[16] Muscat M, Shefer A, Ben Mamou M, Spataru R, Jankovic D, Deshevoy S (2014). The state of measles and rubella in the WHO European region, 2013. Clin Microbiol Infect.

[17] World Health Organization. Regional Office for the Western Pacific. Measles elimination field guide. Manila: WHO Regional Office for the Western Pacific; 2013. https://apps.who.int/iris/handle/10665/207664.

[18] Orenstein WA, Hinman A, Nkowane B, Olive JM, Reingold A. Measles and Rubella Global Strategic Plan 2012-2020 midterm review. Vaccine. 2018;36 Suppl 1:A1–A34. 10.1016/j.vaccine.2017.09.026. PMID: 29307367. 10.1016/j.vaccine.2017.09.026 29307367

[19] Li J, Lu L, Pang X, Sun M, Ma R, Liu D, et al. A 60-year review on the changing epidemiology of measles in capital Beijing, China, 1951-2011. BMC Public Health. 2013;13:986. 10.1186/1471-2458-13-986. PMID: 24143899; PMCID: PMC4016557. 10.1186/1471-2458-13-986 PMC4016557 24143899

[20] Edward S (2015). A mathematical model for control and elimination of the transmission dynamics of measles. Appl Comput Math.

[21] Shorunke FO, Adeola-Musa O, Usman A, Ameh C, Waziri E, Adebowale SA (2019). Descriptive epidemiology of measles surveillance data, Osun state, Nigeria, 2016-2018. BMC Public Health.

[22] Ibrahim BS, Usman R, Mohammed Y, Okunromade O, Abubakar A, Nguku P. Burden of measles in Nigeria: a five-year review of casebased surveillance data, 2012-2016. Pan Afr Med J. 2019;32(Suppl 1):5. 10.11604/pamj.supp.2019.32.1.13564. 10.11604/pamj.supp.2019.32.1.13564 PMC6445333 30984326

[23] Njim T, Agyingi K, Aminde LN, Atunji EF (2016). The trend in mortality from a recent measles outbreak in Cameroon: a retrospective analysis of 223 measles cases in the Benakuma Health District. Pan Afr Med J.

[24] Modu Isa A, Isa AM, Baba AM, Sheriff SA, Isa MA, Author C. Statistical analysis of measles incidence in borno state, nigeria. Available from: https://www.researchgate.net/publication/339272578

[25] Ibrahim BS, Usman R, Mohammed Y, Datti Z, Okunromade O, Abubakar AA (2019). Burden of measles in Nigeria: a five-year review of case-based surveillance data, 2012–2016. Pan Afr Med J.

[26] Obagha C, Gidado S, Uba B, Ajisegiri S, Nguku P, Bamidele F (2018). Surveillance data analysis on measles cases, Anambra state, Nigeria, 2011-2016. Int J Infect Dis.

[27] Fatiregun AA, Adebowale AS, Fagbamigbe AF (2014). Epidemiology of measles in Southwest Nigeria: An analysis of measles case-based surveillance data from 2007 to 2012. Trans R Soc Trop Med Hyg.

[28] Duru CO, Peterside O, Adeyemi OO (2014). A 5-year review of childhood measles at the Niger Delta University teaching hospital, Bayelsa state, Nigeria. J Med Med Sci.

[29] Fatiregun AA, Adebowale AS, Fagbamigbe AF (2014). Epidemiology of measles in Southwest Nigeria: an analysis of measles case-based surveillance data from 2007 to 2012. Trans R Soc Trop Med Hyg.

[30] Mafigiri R, Nsubuga F, Ario AR. Risk factors for measles death: Kyegegwa District, western Uganda, February–September, 2015. BMC Infect Dis. 2017;17:462. 10.1186/s12879-017-2558-7. 10.1186/s12879-017-2558-7 PMC5496348 28673250

[31] Progress towards measles elimination – African Region, 2013–2016. Relev Epidemiol Hebd. 2017;92(18):229–39. 28530366

[32] Kisangau N, Sergon K, Ibrahim Y, Yonga F, Langat D, Nzunza R, et al. Progress towards elimination of measles in Kenya, 2003-2016. Pan Afr Med J. 2018;31. 10.11604/pamj.2018.31.65.16309. 10.11604/pamj.2018.31.65.16309 PMC6457729 31007812

[33] Nsubuga F, Bulage L, Ampeire I, Matovu JKB, Kasasa S, Tanifum P, et al. Factors contributing to measles transmission during an outbreak in Kamwenge District, Western Uganda, April to August 2015. BMC Infect Dis. 2018;18:21. 10.1186/s12879-017-2941-4. 10.1186/s12879-017-2941-4 PMC5759285 29310585

[34] Ibrahim BS, Usman R, Mohammed Y, Datti Z, Okunromade O, Abubakar AA, Nguku PM. Burden of measles in Nigeria: a five-year review of casebased surveillance data, 2012–2016. Pan Afr Med J. 2019;32(Suppl 1):5. 10.11604/pamj.supp.2019.32.1.13564. PMID: 30984326; PMCID: PMC6445333. 10.11604/pamj.supp.2019.32.1.13564 PMC6445333 30984326

[35] Who-Afro (2004). World Health Organization Regional Office for Africa Guidelines for Measles Surveillance, Revised December 2004.

[36] Saleh JA (2016). Trends of measles in Nigeria : A systematic review.

[37] Cutts FT, Claquin P, Danovaro-Holliday MC, Rhoda DA (2016). Monitoring vaccination coverage: defining the role of surveys. Vaccine.

[38] National Bureau of Statistics. Nigeria National Immunization Coverage Survey (NICS): National Brief, Lagos, Nigeria. Available from: https://www.jhsph.edu/ivac/wp-content/uploads/2018/04/Nigeria-NICS-National-Brief.pdf. Accessed 12 Jul 2020.

